# Ameliorative and Synergic Effects of Derma-H, a New Herbal Formula, on Allergic Contact Dermatitis

**DOI:** 10.3389/fphar.2020.01019

**Published:** 2020-07-14

**Authors:** Si Yeon Jo, Mi Hye Kim, Haesu Lee, Sun Haeng Lee, Woong Mo Yang

**Affiliations:** ^1^ Department of Convergence Korean Medical Science, College of Korean Medicine, Kyung Hee University, Seoul, South Korea; ^2^ Department of Clinical Korean Medicine, Graduate School, Kyung Hee University, Seoul, South Korea

**Keywords:** allergic contact dermatitis, *Astragalus membranaceus* Fisch. ex Bunge, *Nepeta tenuifolia* Benth, pruritus, inflammation

## Abstract

Allergic contact dermatitis (ACD) is characterized by itching, skin inflammation, and allergic responses caused by release of immunoglobulin E and T helper 2-specific cytokines. The aim of this study is to investigate the ameliorative and synergic effects of herbal formula, Derma-H, containing *Astragalus membranaceus* Fisch. ex Bunge (AM) and *Nepeta tenuifolia* Benth (NT) which have been used as traditional medicinal herbs for the cure of dryness, edema, and pruritus. 2,4-Dinitrochlorobenzene (DNCB) was applied for ACD induction. AM, NT, and a mixture of AM and NT was topically applied to skin lesions for 11 days. Dermatitis score and number of scratches were significantly diminished in AM, NT, and AM + NT (Derma-H)-treated groups. Especially, Derma-H was more effective than single treatment of AM and NT on skin hyperplasia and mast cell infiltration. Also, NGF expression decreased by NT and a mixture of AM and NT. Additionally, series of TrkA, Raf-1, MEK, and ERK were significantly inhibited by topical AM and NT application. Those findings suggested AM and NT treatment has a synergic effect on DNCB-induced ACD in mice.

## Introduction

Allergic contact dermatitis (ACD), a delayed-type of hypersensitivity, is caused by a variety of contact allergens. Approximately, 7% of worldwide population suffers from ACD and the prevalence rate of ACD is consistently increasing (Kim et al., 2013). ACD patients suffer from several symptoms including relapsing eczema, swelling, redness, dryness, and itching (Lipozencic and Wolf, 2007). There are several factors to trigger ACD such as stress, irritants, allergens, microorganisms, and environmental factors (Park et al., 2016). Although the pathogenesis of ACD is not fully known, multiple studies reported that inflammatory and pruritic mediators are involved in the progress of ACD (Nedoszytko et al., 2014). T helper (Th) 2 cytokines are typical inflammatory mediators in ACD (Kim et al., 2018). In addition, recruitment of nerve growth factor (NGF) and interleukin (IL)-31 induces severe itching in ACD (Feld et al., 2016). Natural killer cells, T regulatory cells, B cells, epidermal Langerhans cells, and keratinocytes are also involved in ACD (Gober and Gaspari, 2008).

Because the incidence of ACD is still increasing, the therapeutic request for improving ACD is gradually rising (Lee et al., 2016). To alleviate ACD symptoms, there are various drugs such as anti-inflammatory, anti-histamine, and glucocorticosteroid drugs as well as moisturizers (Yuan et al., 2010; Lee et al., 2017). However, long period-use of steroids including dexamethasone causes skin weakening, facial edema, psoriasis, furuncles, dryness, and bleeding (Walling and Swick, 2010). For those reasons, ACD patients hesitate to take such drugs because of their severe side effects (Arkwright et al., 2013). Thus, the development of novel ACD treatment using plant-derived natural compounds remains as a global challenge (Kim et al., 2013).

In East Asia, the dried root of *Astragalus membranaceus* Fisch. ex Bunge (AM) has been used as a conventional medicinal herb for more than 2,000 years (Zhou et al., 2018). Additionally, AM has a therapeutic effect on inflammation, skin-reinforcing, wound healing, and immune-regulation (Cho and Leung, 2007). The dried leaves of *Nepeta tenuifolia* Benth (NT) have been widely used in Japan, China, and Korea as an anti-inflammatory treatment for influenza symptoms such as headache, cough, nasal plug, fever, and severe fatigue (Grewe et al., 1998). Clinically, many prescriptions contain AM and NT such as Danggwieumja (Dangguiyinzi), Haedoknaetaksan (Jieduneituosan), Danggwieum (Dangguiyin), Daegosamhwan (Dakushenwan), and Haedokbangpungtang (Jiedufangfengtang) are used to cure skin diseases. Especially, it is reported that AM preserves cutaneous lesions by alleviating the severity of psoriasis, furuncle, and eczema and regenerating of skin tissues, while NT usually improves allergic, inflammatory, and infectious skin disease. Based on the previous studies (Choi et al., 2013; Choi et al., 2016; Choi et al., 2018), the hypothesis was suggested that a mixture of AM and NT has synergic effects on ACD.

In this study, the effects and its possible mechanism of AM and NT on ACD were investigated in DNCB-induced mice model. Histopathological features of skin lesions and scratching behaviors were analyzed. Especially, to demonstrate the clinical efficacy of AM and NT on pruritus, NGF, Tropomyosin receptor kinase A (TrkA), Raf-1 (Serine/Threonine kinase), MEK (MAPK/ERK kinase), and Extracellular signal-regulated kinases (ERK) pathway and interleukin (IL)-31 were examined. Moreover, expression levels of interleukin (IL)-4, -6, -10, -13, tumor necrosis factor** (**TNF)-α, and transforming growth factor** (**TGF)-β in ACD-like skin lesions were confirmed the inhibitory effects of AM and NT on immune responses in ACD.

## Materials and Methods

### Chemicals and Reagents

2,4-dinitrochlorobenzene (DNCB) and Dexamethasone was purchased from Sigma Aldrich (St.Louis, USA). Sodium dodecyl sulfate (SDS) was applied by Biosesang (Sungnam, Korea). Antibodies used in Western blot such as β-actin, Raf-1, p-Raf-1, anti-rabbit, and anti-mouse horseradish peroxidase-conjugated secondary antibody were obtained from Santa Cruz (CA, USA). ERK, p-ERK, MEK, p-MEK were purchased from Cell Signaling Technology (Danvers, MA, USA). In addition, antibody of TrkA was applied by Abcam (Cambridge, UK). For reverse transcription polymerase chain reaction (RT-PCR) experiment, Maxime RT Premix and Maxime PCR premix were purchased from iNtRON Biotechnology Inc. (Sungnam, Korea). Specific primers such as IL-4, -6, -10, -13, -31, TNF-α, interferon (IFN)-γ, TGF-β, and GAPDH used for amplifying were designed by Bioneer Corp. (Daejeon, Korea).

*Preparation of Sample A. membranaceus* Fisch. ex Bunge and N*. tenuifolia* Benth, which are originated from Korean medicine, were obtained from Dong-Yang Herb (Seoul, Korea). The batch numbers of *A. membranaceus* Fisch. ex Bunge and N*. tenuifolia* Benth were DY074K02H and DY052S02H. Ten grams of AM and NT, respectively, were extracted with 100 ml of distilled water by refluxing at 100°C for 2 h. The extracts were filtered by vacuum filter and condensed in a rotary evaporator under reducing pressure. Then condensed samples were pre-dried in −80°C about 48 h. Samples were powdered under freeze-dryer for 72 h. The yields of AM and NT were 29.57 and 17.10%.

### Standardization of AM and NT

Each of AM and NT extracts were identified by formononetin and pulegone using high-performance liquid chromatography diode array detector (HPLC-DAD). Formononetin and pulegone were chosen to identify AM and NT extracts, respectively, as standard compounds according to the National Standard of Traditional Medicinal (Herbal and Botanical) Materials in the Korean Pharmacopoeia. The HPLC apparatus was a Gilson System equipped with a 234 Autosampler, a UV/VIS-155 detector and a 321 HPLC Pump (Gilson, Seoul, Korea). In case of AM, chromatography was performed at room temperature at a flow rate of 0.5 ml/min and 10 μl was analyzed for 50 min. The retention time of formononetin was 31.7 min. Standardization of NT was based on the content of pulegone. Chromatographical analysis was performed at room temperature at a flow rate of 1.0 ml/min, and 10 μl was analyzed for 30 min. The retention time of pulegone was 18.4 min. Consequently, the concentrations of formononetin and pulegone were 2 mg/g in AM extract and 7.03 mg/g in NT extract, respectively (**Figures S1** and **S2**).

### Animals

All of 30 male BALB/c mice aged 5-week-old were purchased from Raonbio Inc. (Yongin, Korea). Animals were housed in a well-controlled animal room with a 12 h light/dark cycle and humid condition of 50 ± 5% at 22–24°C. All mice were fed with standard chow diet and tap water. Animal care and experimental procedures were performed in accordance with the “Guide for the Care and Use of Laboratory Animals” (Department of Health, Education and Welfare, NIH publication #78-23, 1996). This animal experiment was approved by the Guide for the Care and Use of Laboratory Animals of Kyung Hee University, Seoul, Korea (KHUASP(SE)-16-159).

After 1 week of adaptation, mice were divided into six groups (n = 5): (i) NOR: normal control, with no sensitization and vehicle application; (ii) DNCB: negative control, with DNCB sensitization and vehicle application; (iii) DEX: positive control, with DNCB sensitization and 10 μM (3.9246 × 10^-3^ mg/ml) of dexamethasone application; (iv) AM: with DNCB sensitization and 30 mg/ml of AM application; (v) NT: with DNCB sensitization and 20 mg/ml of NT application; and (vi) AM + NT: with DNCB sensitization and 50 mg/ml AM and NT, 30 mg AM, and 20 mg NT in 1 ml distilled water, application. The concentrations of AM (30 mg/ml) and NT (20 mg/ml) were determined by each yield to correspond to the same amount of raw materials. A mixture consists of 30 mg of AM and 20 mg of NT dissolved in 1 ml distilled water. Animal experiment was performed as described previously (Choi et al., 2017). Before the experiment, the skin on the upper back was shaved by an electronic shaver. 2,4-dinitrochlorobenzene (DNCB) was used as a sensitization substance for ACD, which initiates skin innate immunity activation. For first challenge of ACD, 200 μl of 1% DNCB in acetone/olive oil (4:1, v/v) was topically applied to the dorsal skin of mice once daily. After 3 days of sensitization, mice did not receive any further treatment during the next 4 days. Then, sodium dodecyl sulfate (SDS), samples, and DNCB were treated every 3 h in order, respectively, at second challenge of ACD. In detail, 200 μl of 4% SDS was applied to the dorsal skin of mice to disrupt the skin barrier and remove cuticle layers. After 3 h, 200 μl of samples including AM, NT, and mixture were topically administrated to the dorsal skin, while DNCB group was received vehicle. Then after 3 h of sample treatment, 200 μl of 0.5% DNCB was treated to the skin lesions of mice. All treatments including 4% SDS, samples, and 0.5% DNCB were repeated for consecutive 11 days. There was no side effects or abnormalities following AM and NT treatment during the experiment. At the end of experiment, all mice were sacrificed.

### Measurement of Dermatitis Score

The extent of (1) erythema/hemorrhage, (2) dryness/scarring, (3) edema, and (4) erosion/excoriation was scored as 0 (none), 1 (mild), 2 (moderate), or 3 (severe). All mice were photographed by digital camera (Sony, Tokyo, Japan) under anesthesia. The severity of the dorsal skin was measured by three researchers without group information. The sum of the separate scores was described as the dermatitis score. Before sacrifice, mice were anesthetized with 100 μl of zoletil and rompun mixture. Then the severity of the dorsal skin in all mice was measured by triplicate.

### Measurement of Scratching Behavior

The previous day of sacrifice, all mice were set in a separated plastic cage for stabilizing for 1 h after sensitization with 200 μl of 0.5% DNCB. Scratching behavior of each group was recorded for 20 min. The number of scratching was counted by acknowledging the scratches only the back side of the body. The scratching episode of each group was counted by a blind test. The number of scratching of each mouse was averaged from the counts of three researchers.

### Histological Analysis for H&E Staining

Skin tissues from the upper back of mice were fixed in 4% formalin for 24 h, dehydrated. After embedding of each specimen, the specimens were sliced to 4 μm of thickness. Sections of skin specimens were stained with hematoxylin and eosin (H&E). Stained specimens were cover-slipped with VECTA Mount permanent mounting medium (VECTOR laboratories, Inc., Burlingame, CA). Digital images were obtained from Leica Application Suite (LAS) Microscope Software (Leica Microsystems Inc., IL, USA). The magnification of H&E staining was ×100.

### Histological Analysis for Toluidine Blue Staining

For the evaluation of mast cell infiltration, sliced specimens of 4 μm thickness were stained with toluidine blue solution. Digital images were obtained from Leica Application Suite (LAS) Microscope Software. Then five sites of every stained specimen were chosen for counting mast cells numbers at random with the scale of ×200.

### Western Blot Analysis

Each sample of dorsal skins were homogenized in tissue protein extraction buffer (Thermo scientific, Rockford, USA) with protease inhibitor cocktail tablets (Roche, Mannheim, Germany). Twenty micrograms of protein from dorsal skin was denatured with 5% SDS loading buffer. The prepared samples were loaded on 10% SDS-polyacrylamide gel electrophoresis. Then electro-transferred to activated polyvinylidene fluoride membranes. Membranes were blocked by 3% bovine serum albumin in tris-buffered saline (TBS) containing 1% tween 20 (TBS-T) and incubated with the specific antibodies at 4°C overnight (β-actin, Raf-1, p-Raf-1; Santa Cruz, CA, USA, 1:1,000 dilutions in TBS-T, ERK, p-ERK, MEK, p-MEK; Cell Signaling Technology, Danvers, MA, USA, 1:1,000 dilution in TBS-T, TrkA; Abcam, Cambridge, UK, 1:1,000 dilution in TBS-T). After washing of those membranes for 10 min three times, membranes were incubated with anti-rabbit and anti-mouse horseradish peroxidase-conjugated secondary antibody (Santa Cruz, 1:2,000 dilutions in TBS-T) for 1 h at room temperature. Then proteins were developed with an enhanced chemiluminescence detection reagent (AbClon, Seoul, Korea) by a Davinch-Western imaging system (Davinch-K, Seoul, Korea).

### Measurement of Inflammatory Cytokine Levels

Total RNA from dorsal skin lesions was extracted with RNA extraction solution (Nanohelix, Daejeon, Korea) following the manufacturer’s instruction. Isolated total 2 μg RNA was synthesized into cDNA using Maxime RT Premix (iNtRON Biotechnology Inc., Sungnam, Korea). Synthesis of cDNA was arranged as follows: stage 1: 45°C for 60 min; stage 2: 95°C for 5 min. The cDNA was amplified with using Maxime PCR premix (iNtRON Biotechnology Inc., Sungnam, Korea) and specific primers such as IL-4, -6, -10, -13, -31, TNF-α, interferon (IFN)-γ, TGF-β, and GAPDH. The primers used for amplifying were designed by Bioneer Corp. (Daejeon, Korea) and listed in **Table 1**. Optimal conditions of each factors for RT-PCR were confirmed by triplicate. Gene expressions were relatively estimated with the housekeeping genes (GAPDH) mRNA levels. The relative changes of target gene expression were analyzed by Gel doc (DAIHAN Scientific Co, Ltd., Wonju-si, Gangwon-do, Korea).

**Table 1:** Sequence of reverse transcription PCR primers.

Target gene	5′ → 3′ Forward primer	5′ → 3′ Reverse primer
IL-6	CGGAGAGGAGACTTCACAGAGGA	GGAGAGCATTGGAAATTGGGG
TNF-α	CCTGTAGCCCACGTCGTAGC	TTGACCTCAGCGCTGAGTTG
IL-10	GACCAGCTGGACAACATACTGCTAA	GTTGAGATGATGCTTTGACAGAAG
TGF-β	CGGGGCGACCTGGGCACCATCCATGAC	CTGCTCCACCTTGGGCTTGCGACCCAC
IL-4	ATGGGTCTCAACCCCCAGC	GCTCTTTACGCTTTCCAGGAAGTC
IL-13	ACCACGGTCATTGCTCTCA	GTGTCTCGGACATGCAAGCT
IL-31	TCGGTCATCATAGCACATCTGGAG	GCACAGTCCCTTTGGAGTTAAGTC
IFN-γ	AGCGGCTGACTGAACTCAGATTGTAG	GTCACAGTTTTCAGCTGTATAGGG
GAPDH	GGCATGGACTGTGGTCATGA	TTCACCACCATGGAGAAGGC

### Statistical Analysis

Data are presented as the mean ± standard error of the mean (S.E.M). Differences between control groups and application groups were examined using a one-way analysis of variance (ANOVA) and Tukey’s tests. *p* < 0.05 were considered statistically significant.

## Results

### Effect of AM and NT on Skin Dermatitis Score and Scratching Behavior

**Figure 1:**
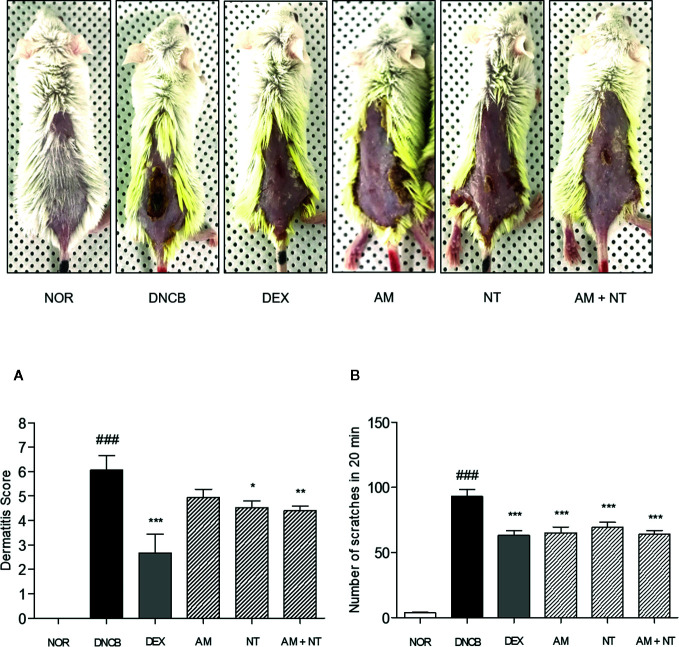
Effects of AM, NT, and a mixture of AM and NT on skin dermatitis scores **(A)** and scratching behaviors **(B)** with allergic contact dermatitis skin lesions. The scratching behavior was evaluated 1 h after the last DNCB application on the day before sacrifice. Data were presented as means ± S.E.M (n = 5). ^###^ *p* < 0.001 *vs.* NOR; ^*^ *p* < 0.05, ^**^ *p* < 0.01, and ^***^ *p* < 0.001 *vs.* DNCB.

Dermatitis symptoms including erythema, dryness, edema, erosion were developed following the DNCB sensitization. As a negative control; dermatitis score of DNCB group was calculated about 6.1, unlike 0 of the normal control group; NOR group. Compared with DNCB group, skin severity in DEX group as a positive control was clearly ameliorated as shown in 56.0% reduction of dermatitis score. The skin severity of each group were improved as following the application of AM and NT. Compared with DNCB group, Dermatitis scores of AM and NT group were decreased by 18.7% and 25.3%, respectively. Moreover, AM + NT group also showed noticeable improvement of skin severity. The score of AM + NT group was decreased by 27.5% compared with DNCB group (**Figure 1A**).

Additionally, DNCB-treated mice showed a noticeable increase of scratching actions than NOR group about 24.8 folds. As a positive control, DEX treatment depressed the number of scratching behavior up to 32.1% compared with DNCB group. Each of AM and NT treatment ameliorated the scratching behavior up to 30.0 and 25.6%. In addition, topical application of AM and NT reduced the scratching behavior up to 31.2% compared with DNCB group (**Figure 1B**).

### Effect of AM and NT on Histological Changes

**Figure 2:**
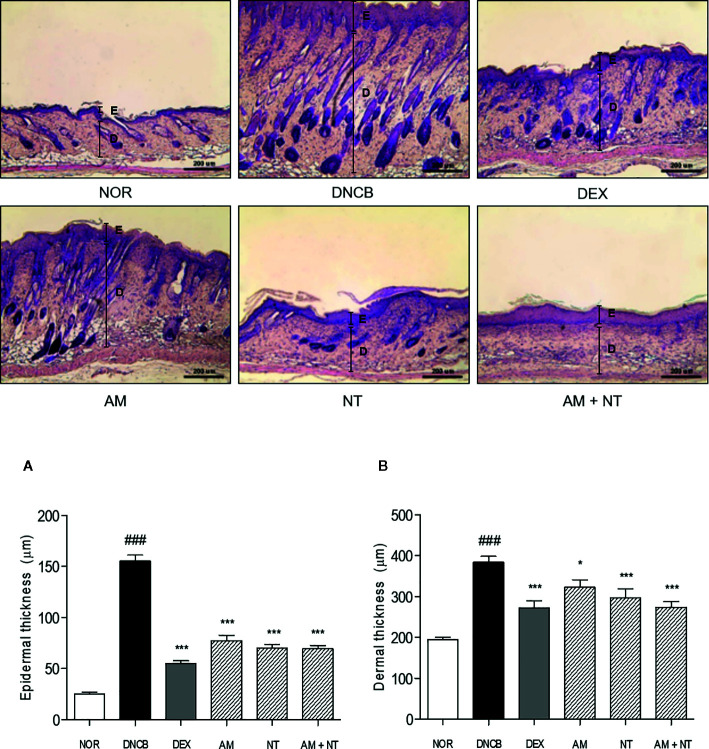
Effects of AM, NT, and a mixture of AM and NT on histopathological features with allergic contact dermatitis skin lesions. Epidermis **(A)** and dermis **(B)** thickness were stained with hematoxylin and eosin and measured microscopically. The magnification was ×100. Data were presented as means ± S.E.M (n = 5). ^###^ *p* < 0.001 *vs.* NOR; ^*^ *p* < 0.05 and ^***^ *p* < 0.001 *vs.* DNCB. Black lines indicate the layer of epidermis (E) and dermis (D).

As follow H&E staining, the DNCB group indicated skin hyperplasia of the epidermis and lichenification. Thickness of epidermis and dermis were increased 6.1- and 2.1 folds in DNCB group compared with NOR group. There were significant reductions of epidermal and dermal thickness in all treatment groups compared with DNCB group. Each AM and NT treatment influenced skin hyperplasia compared with DNCB group (Epidermis: AM 50.1%, NT 54.7%; dermis: AM 18.9%, NT 31.6%). Similarly, the treatment of mixture, Derma-H, markedly inhibited hyperplasia of the epidermis and dermis similar to DEX (Epidermis: AM + NT 55.2%, DEX 64.4%; dermis: AM + NT 35.2%, DEX 25.8%) (**Figures 2A, B**).

### Effect of AM and NT on Mast Cell Degranulation

**Figure 3:**
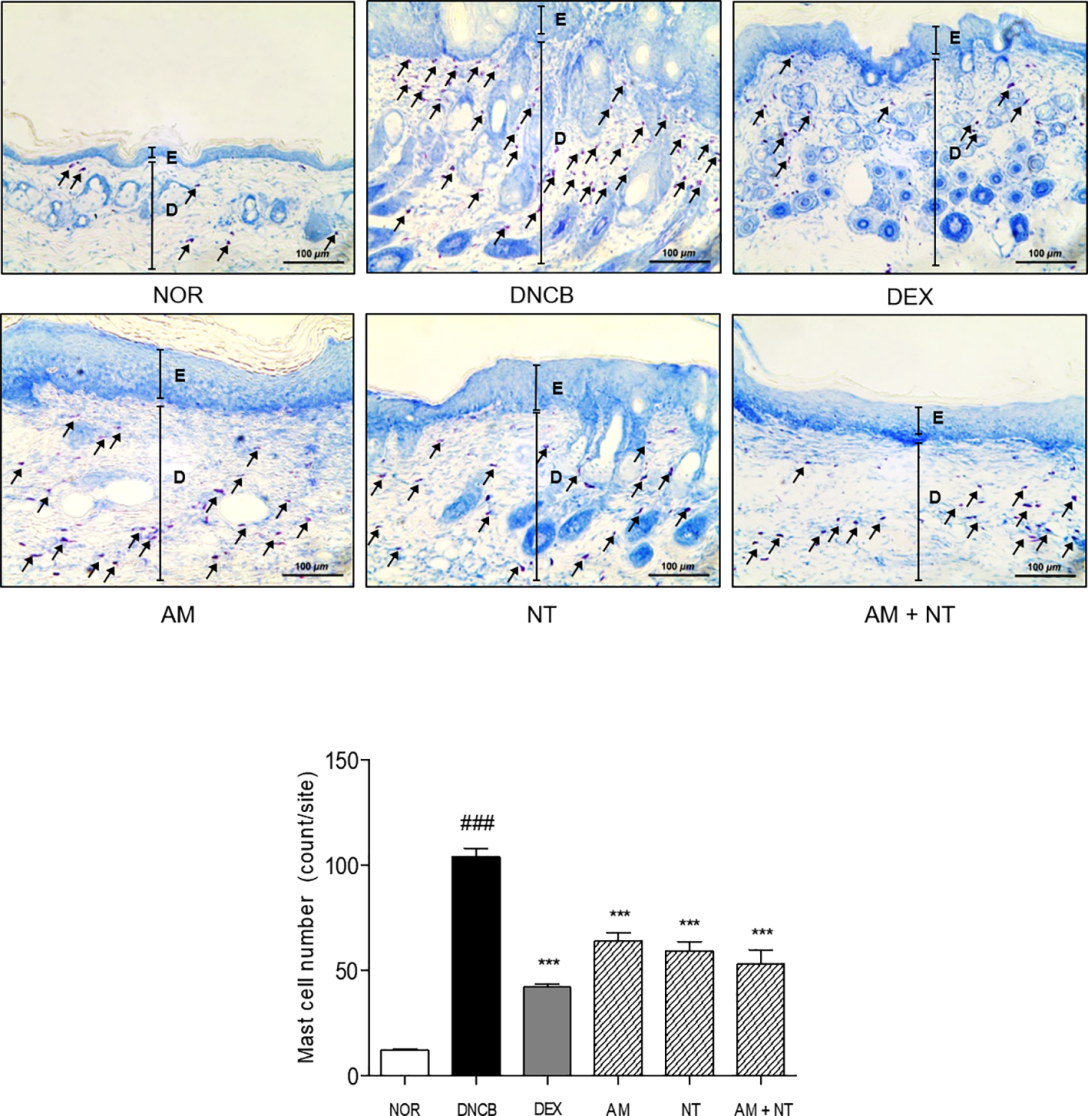
Effects of AM, NT, and a mixture of AM and NT on mast cell degranulation with allergic contact dermatitis skin lesions. Mast cell infiltration in dorsal skin lesions were indicated by Toluidine blue staining. The magnification is ×200. Data were presented as means ± S.E.M (n = 5). ^###^ *p* < 0.001 *vs.* NOR; ^***^ *p* < 0.001 *vs.* DNCB. Black lines indicate the layer of epidermis (E) and dermis (D). Arrows indicate the toluidine blue-stained mast cells.

As follow toluidine blue staining, the infiltration of mast cells in DNCB group was 8.7 folds higher than that in NOR group. Each treatment of AM and NT also reduced mast cell infiltration about 38.5 and 43.0%, respectively. In addition, the number of mast cells was significantly reduced by 49.0% in AM and NT treated mice (**Figure 3**).

### Effect of AM and NT on NGF-TrkA Signaling Pathway-Related Factors

**Figure 4:**
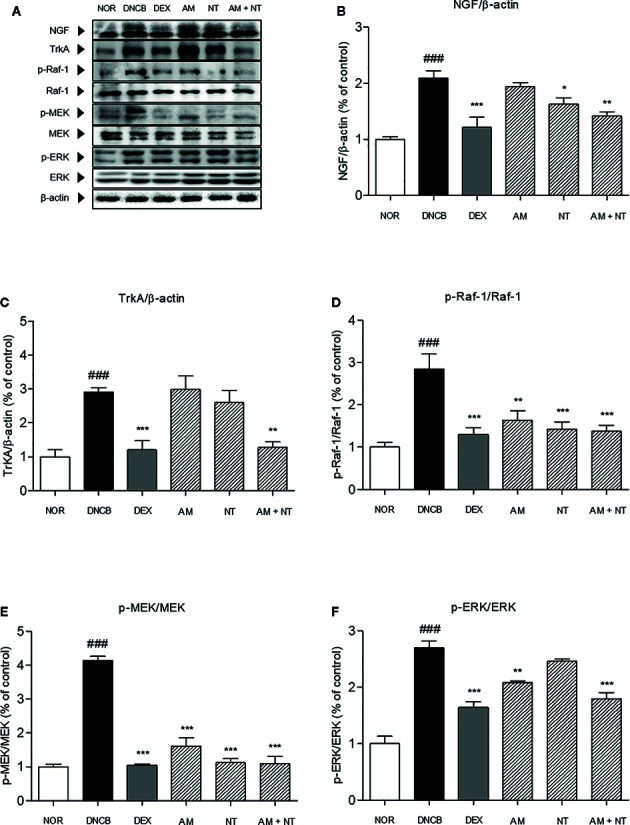
Effects of AM, NT, and a mixture of AM and NT on NGF-TrkA signaling related factors with allergic contact dermatitis skin lesions. **(A)** The expressions of NGF-TrkA signaling related factors, such as **(B)** NGF, **(C)** TrkA, **(D)** Raf-1, **(E)** MEK, and **(F)** ERK were determined on the dorsal skin by Western blot analysis. Data were presented as means ± S.E.M (n = 5). ^###^ *p* < 0.001 *vs.* NOR; ^*^ *p* < 0.05, ^**^ *p* < 0.01, and ^***^ *p* < 0.001 *vs.* DNCB.

To identify the pruritic effects of AM and NT mixture, the expressions of NGF-TrkA signaling pathway-related factors were estimated (**Figure 4A**). Expression of NGF in DNCB group was 2.1 folds higher than NOR group. Topical treatment of DEX reduced NGF expression about 42.0% compared with DNCB group. Compared with DNCB group, the expressions of NGF in AM and NT treated groups were reduced about 11.8 and 16.3% respectively. Similar to the result of DEX group, a mixture of AM and NT down-regulated the expression of NGF up to 32.5% (**Figure 4B**).

Series of TrkA, Raf-1, MEK, and ERK levels were analyzed by Western blot analysis. Phosphorylation of TrkA, Raf-1, MEK, and ERK were considerably increased by 2.9, 2.8, 4.1, and 2.7 folds in DNCB-induced ACD skin, compared with NOR group. With the treatment of AM and NT, series of TrkA, Raf-1, MEK, and ERK expressions were remarkably inhibited by 56.2, 77.6, 73.6, and 33.5% compared with DEX group about 58.7, 22.6, 74.8, and 39.3% (**Figures 4C–F**). Also, Raf-1, MEK, and ERK phosphorylation were decreased in AM and NT groups, respectively (Raf-1: AM 42.5%, NT 50.0%; MEK: AM 61.0%, NT 72.8%; ERK: AM 6.5%, NT 15.7%).

### Effect of AM and NT on Inflammatory Cytokines

**Figure 5:**
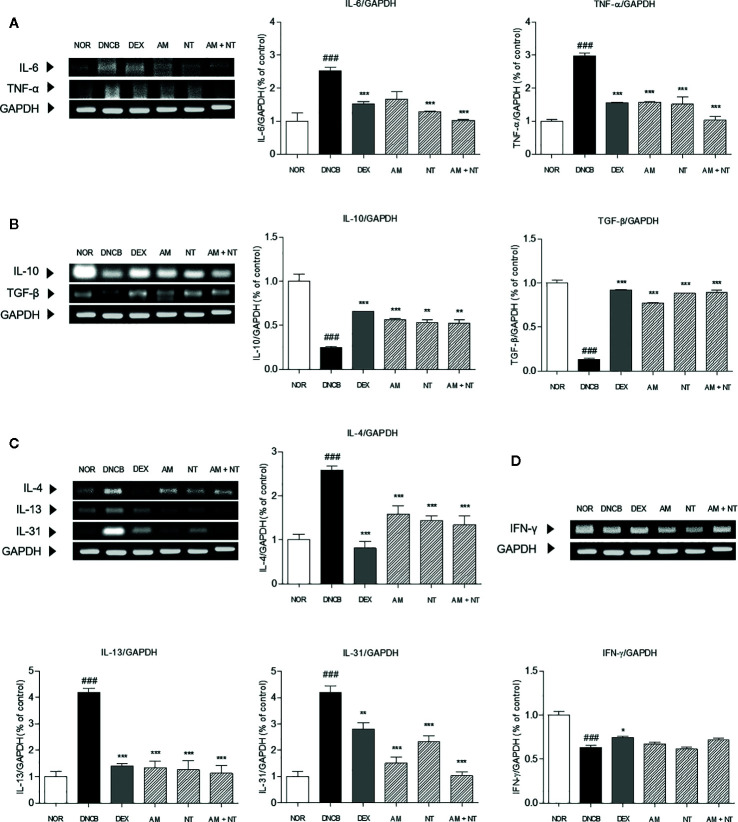
Effects of AM, NT, and a mixture of AM and NT on inflammatory cytokine levels: **(A)** pro-inflammatory cytokines; interleukin (IL)-6 and TNF-α, **(B)** anti-inflammatory cytokines; IL-10 and TGF-β, **(C)** Th2 specific cytokines; IL-4, -13, and -31; and **(D)** Th1 specific cytokines; IFN-γ in mice with allergic contact dermatitis skin lesions. Data were presented as means ± S.E.M (n = 5). ^###^ *p* < 0.001 *vs.* NOR; ^*^ *p* < 0.05, ^**^ *p* < 0.01, and ^***^ *p* < 0.001 *vs.* DNCB.

To investigate the anti-inflammatory effects of AM and NT, mRNA expression levels of inflammatory-mediators including pro-inflammatory cytokines (IL-6 and TNF-α), counter-regulatory cytokines (IL-10 and TGF-β), Th2 cytokines (IL-4, -13, and -31), and Th1 cytokine (IFN-γ) in the dorsal skin was analyzed by RT-PCR. DNCB group up-regulated the expressions of pro-inflammatory cytokines and Th2-specific cytokines while down-regulated the counter-regulatory cytokines. The recruitment of pro-inflammatory cytokines including IL-6 and TNF-α were increased about 2.5 and 3.0 folds compared with NOR group. Anti-inflammatory cytokines including IL-10 and TGF-β down-regulated about 0.2 and 0.1 folds in DNCB group. Each of AM and NT treatment have regulatory effects on pro-inflammatory cytokines and anti-inflammatory cytokines with the decreases of IL-6 and TNF-α (IL-6: AM 33.9%, NT 48.6%; TNF-α: AM 47.0%, NT 49.0%) (**Figure 5A**) and the increases of IL-10 and TGF-β (IL-10: AM 56.6%, NT 53.8%; TGF-β: AM 82.7%, NT 84.9%) (**Figure 5B**). Compared with DNCB group, topical treatment of AM and NT diminished the levels of IL-6 and TNF-α about 59.3 and 65.3% and up-regulated IL-10 and TGF-β about 53.1 and 85.0%.

Additionally, the expression levels of IL-4, -13, and -31 in DNCB group were 2.6, 4.2, and 4.1 folds higher than NOR group. The expressions of IL-4, -13, and -31 were significantly decreased in AM, NT, and AM + NT groups (IL-4: AM 38.4%, NT 44.1%, AM + NT 48.0%; IL-13: AM 68.2%, NT 69.9%, AM + NT 73.1%; IL-31: AM 64.0%, NT 44.7%, AM + NT 75.5%) (**Figure 5C**). However, Th1-specific cytokine IFN-γ did not appear any increase in AM, NT, and AM + NT groups (**Figure 5D**).

## Discussion

ACD is an inflammatory skin disease associated with skin barrier disruption, leading to intense itching and typically characterized by hyperkeratosis, skin thickening, dryness, excoriation, redness, scars, and edema (Grewe et al., 1998; Napolitano et al., 2016). Dermatitis scores, indicated by erythema/hemorrhage, dryness/scarring, edema, and erosion/excoriation, were improved by AM, NT, and a mixture of AM and NT. Through the histological analysis, skin hyperplasia and swelling were appeared in DNCB group and those symptoms were alleviated by treatment of AM and NT. Single administration of AM and NT, respectively, recovered the characteristics of ACD, such as erythema, hemorrhage, dryness, scarring, erosion, and skin hyperplasia. AM and NT had similar or better inhibitory effects on the development of ACD.

About 60% of ACD patients, especially children, have experienced sleep disturbance regardless of clinical treatment (Fishbein et al., 2018). Because scratching aggravates dermatitis, the susceptibility to itching is increased, resulting in the degeneration of skin lesions (Koblenzer, 1999; Feld et al., 2016). Therefore, the regulation of pruritus and scratching behaviors can be a new therapeutic method for treating ACD (Choi et al., 2018). As described in the previous studies (Choi et al., 2016), topical AM treatment significantly inhibited the scratching behavior by 30.0%. In addition, the number of scratches were decreased at the ratio of 31.2% by treatment of AM and NT, while topical NT application reduced the itching by 25.6%.

There are several pathways to cause scratching and itching in ACD patients such as histamine 1 and 4 receptor, neuropeptides, cytokines, and various inflammatory factors like NGF and mast cell degranulation (Stander and Luger, 2010). Mast cell is the first defender to protect cellular barriers in the cutaneous lesions, the mucous membrane, and digestive and respiratory tracts. Mast cells have been not only associated with acute and chronic responses but also featured by inflammation (Metz et al., 2007). It is well established that the number of mast cells was markedly increased in ACD-like skin lesions (Kawakami et al., 2009). The number of mast cells was remarkably ameliorated in AM, NT, and AM + NT groups as compared with DNCB group.

In association with the mast cells activation, NGF and its receptor (TrkA) have been considered to act as a leading part in the pruritus by inducing “itch-scratch cycle” (Roblin et al., 2015). NGF is a significant neuropeptide that activates nervous system in central and peripheral parts. In addition, NGF correlated inflammatory responses by mast cells, B cells, T cells, neutrophils, and eosinophils (Kritas et al., 2014). TrkA is one of NGF receptors and have a binding with NGF, as a strong affinity nerve growth factor receptor (Mahadeo et al., 1994). This membrane-bounded receptor auto-phosphorylated upon neurotrophin binding (Joca et al., 2015). Activated TrkA triggers Raf-1 to be phosphorylated. Activation of MEK, as a result from phosphorylation by Raf-1, phosphorylates ERK (Roblin et al., 2015). In this study, topical NT and a mixture of AM and NT treatment significantly decreased the expressions of NGF in ACD-like skin lesions, while AM did not affect the NGF expression. The expression of TrkA was markedly diminished in AM and NT treated skin tissues. In consistent with the NGF-TrkA expression, the phosphorylation of series of Raf-1, MEK, and ERK was markedly inhibited in AM, NT, and a mixture of AM and NT treated ACD-like lesions compared with DNCB group. Treatment of AM and NT appeared to be more effective in NGF-TrkA pathway than that of NT.

In addition to itchy lesions, Th cell immune responses are clinical features in ACD (Biedermann et al., 2015). The production of IL-6 and TNF-α initiates the pro-inflammatory reaction in acute stage of ACD. Additionally, counter-regulatory cytokines such as IL-10 and TGF-β are down-regulated by DNCB sensitization, indicating that IL-10 and TGF-β are regarded as anti-inflammatory cytokines. Furthermore, Th2 cells mainly mediate the onset of ACD, while Th1 cells are predominant in the chronic phase of ACD (Leung et al., 2004). Imbalanced Th2/Th1 cell-mediated cytokine is one of critical causes of developing ACD (Racke et al., 1994). IL-4, IL-13, and IL-31 are typical Th2-mediated cytokines in the progress of ACD development (Neis et al., 2006). In contrast, Th1 cells activation increases the release of IFN-γ (Bradley et al., 1996). Especially, Th2-specific cytokines promotes the degranulation of mast cells, that leads to itching sensation. Therefore, inhibition of Th2-mediated cytokines is expected to ameliorate the scratching behavior in ACD. In this study, treatment of AM, NT, and a mixture of AM and NT inhibited the production of pro-inflammatory cytokines, IL-6 and TNF-α, along with increases of anti-inflammatory cytokines, IL-10 and TGF-β. Th2-specific cytokines including IL-4, -13, and -31 were down-regulated, while Th1-specific cytokine IFN-γ was not affected by AM, NT, and a mixture of AM and NT. These results demonstrate that AM and NT had beneficial effects on the down-regulation of Th2-mediated cytokines rather than the up-regulation of Th1-mediated cytokine. Interestingly, the degree of itch score correlates with the IL-31 cytokine production in ACD lesions (Neis et al., 2006). AM treatment showed lower IL-31 expression (64.2%) than NT treatment (44.7%). Although AM did not affect the NGF-TrkA signaling, itching-related cytokine IL-31 was inhibited by AM treatment, resulting in a decrease of scratching behavior.

In this experiment, AM and NT had anti-inflammatory effect on Th and mast cells in clinical phase of ACD. Moreover, they had regulatory effect on keratinocyte which has a role in initiation and termination phase of ACD (Gober and Gaspari, 2008). Astragaloside IV from AM stimulated proliferation and migration of keratinocytes *via* regulation of the Wnt signaling pathway (Li et al., 2012). And NT promoted keratinocyte migration and wound healing (Isohama, 2014).

Epidermal Langerhans cells and dermal dendritic cells may regulate an immune response by presenting allergens to T cells in the paracortical areas of regional lymph nodes in elicitation phase of ACD (Gober and Gaspari, 2008). Further studies confirming the effect of AM or NT on epidermal Langerhans cells or dermal dendritic cells will clearly reveal the mechanism for improving allergic contact dermatitis.

**Figure 6:**
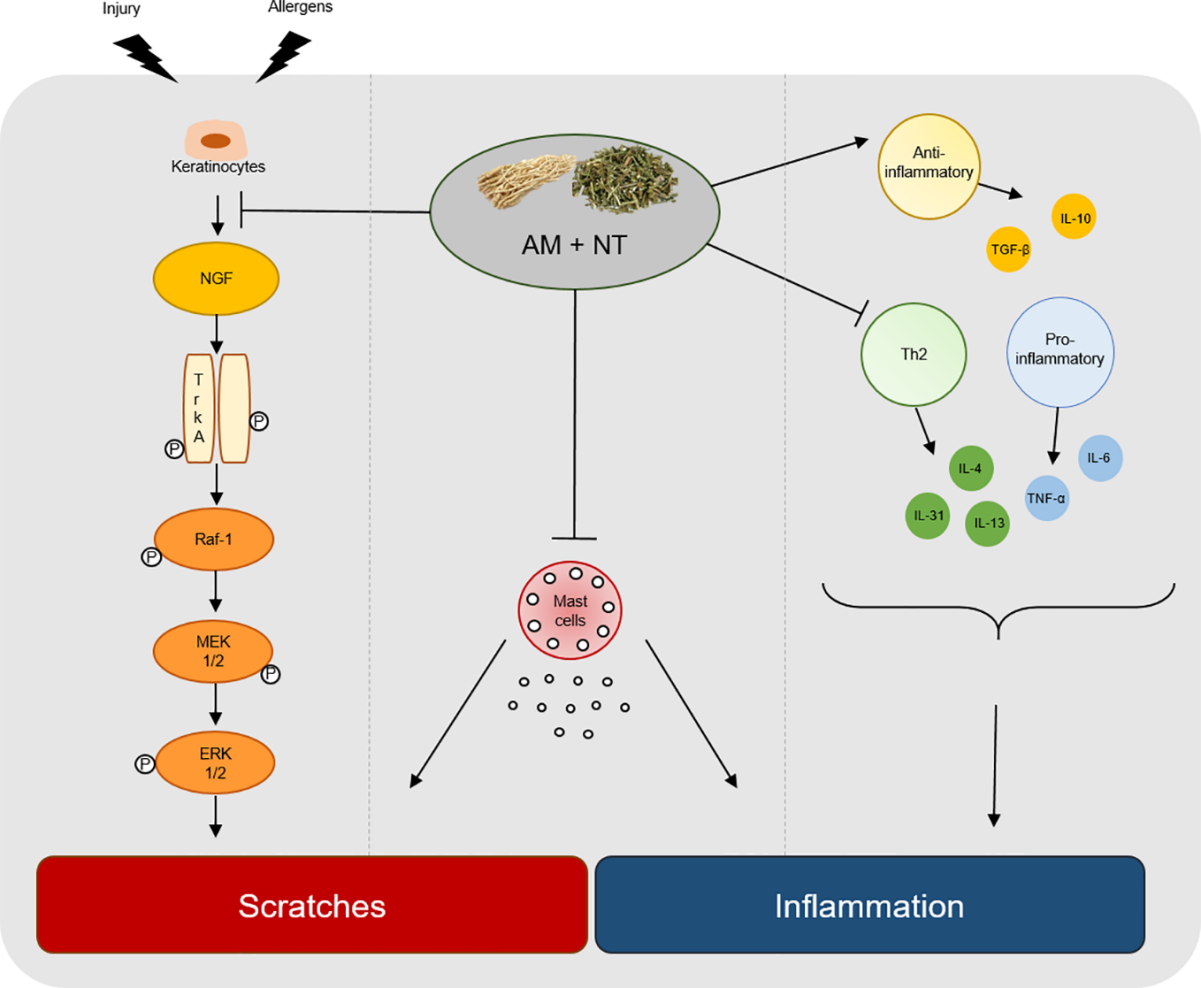
Graphical summary of key process by a mixture of AM and NT, Derma-H, in ACD. Derma-H, a mixture of AM and NT, improved scratches by inhibiting the release of NGF-TrkA signaling and decreasing the number of mast cells. In addition, pro-inflammatory, Th2-specific cytokines and anti-inflammatory cytokines were effectively regulated by Derma-H. Consequently, Derma-H has ameliorative effects on ACD.

Taken together, AM showed the anti-inflammatory effect by decreasing the expression of Th2-mediated cytokines. In addition, NT inhibited NGF-TrkA signaling as an anti-pruritic effect on DNCB-induced skin lesions. In conclusion, a mixture of AM and NT has a synergic effect on pruritus and inflammation in DNCB-induced ACD (**Figure 6**). It may be hypothesized that AM and NT may be further developed as a new alternative therapy for ACD once further experimental work has clarified the concentration of treatment and various clinical effect.

## Data Availability Statement

All datasets generated for this study are included in the article/**Supplementary Material**.

## Ethics Statement

The animal study was reviewed and approved by the Guide for the Care and Use of Laboratory Animals of Kyung Hee University, Seoul, Korea (KHUASP(SE)-14-030).

## Author Contributions

All authors contributed to the article and approved the submitted version. SJ and WY contributed to analysis design. SJ, MK, HL, and SL analyzed data. SJ and WY drafted the manuscript. WY provided supervision of study.

## Funding

This research was supported by a grant of the Korea Health Technology R&D Project through the Korea Health Industry Development Institute (KHIDI), funded by the Ministry of Health & Welfare, Republic of Korea (grant number: HI19C0264).

## Conflict of Interest

The authors declare that the research was conducted in the absence of any commercial or financial relationships that could be construed as a potential conflict of interest.

## Supplementary Material

Figure S1 Standardization of *Astragalus membranaceus* Fisch. ex Bunge using high-performance liquid chromatography (HPLC) systems. HPLC chromatograms of external standard formononetin **(A)** and AM **(B)**.

Figure S2 Standardization of *Nepeta tenuifolia* Benth using high-performance liquid chromatography (HPLC) systems. HPLC chromatograms of external standard pulegone **(A)** and NT **(B)**.
