# Supplementary figures and images for: Ameliorative and Synergic Effects of Derma-H, a New Herbal Formula, on Allergic Contact Dermatitis
Source: Front Pharmacol. 2020 Jul 14;11:1019. doi: 10.3389/fphar.2020.01019 (PMC7371928; doi:10.3389/fphar.2020.01019)

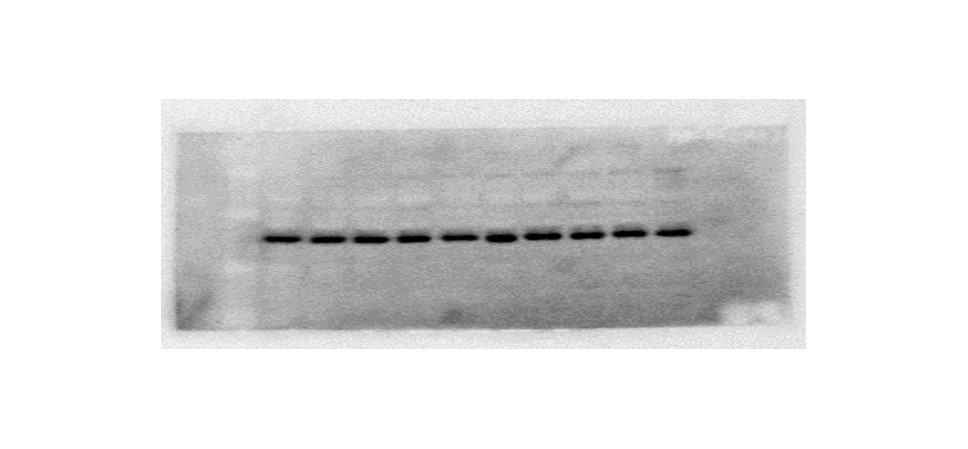

Supplement: Supplementary file 1 [file DataSheet_1.zip › b-actin.jpg]

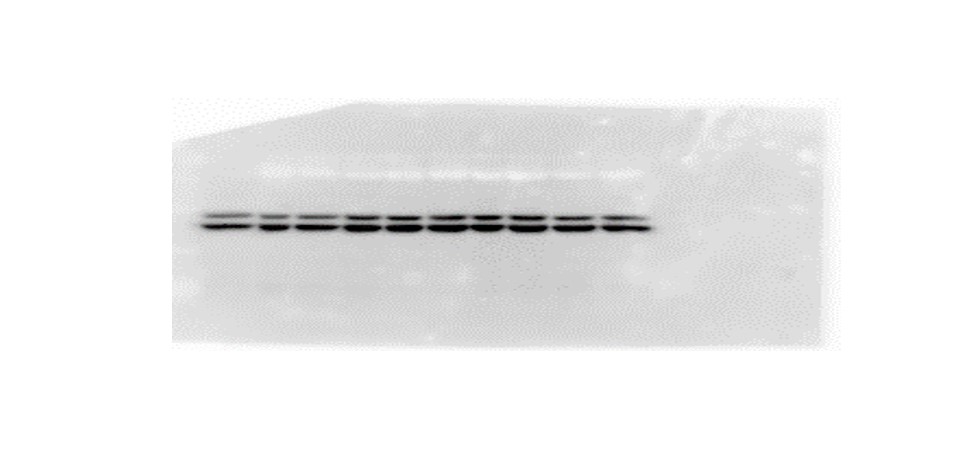

Supplement: Supplementary file 1 [file DataSheet_1.zip › ERK.jpg]

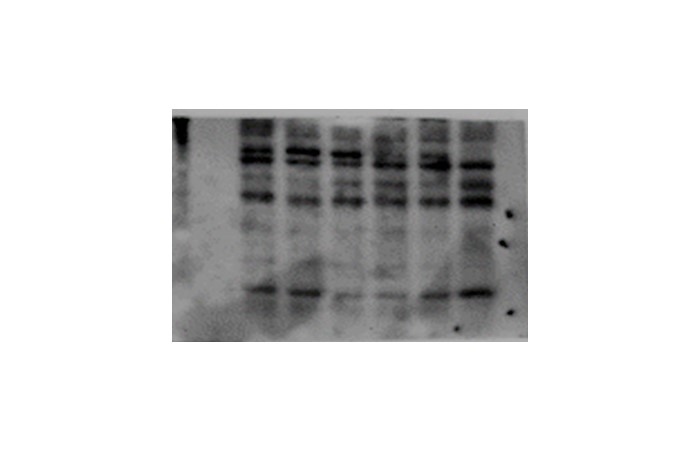

Supplement: Supplementary file 1 [file DataSheet_1.zip › MEK.jpg]

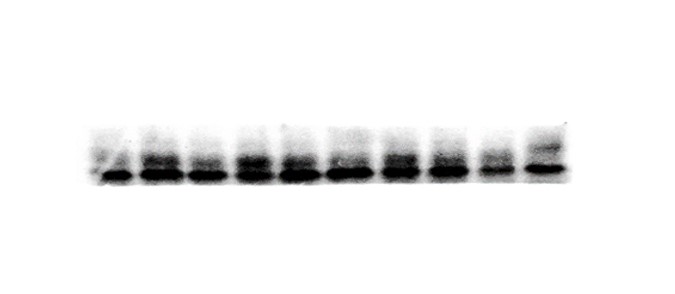

Supplement: Supplementary file 1 [file DataSheet_1.zip › NGF.jpg]

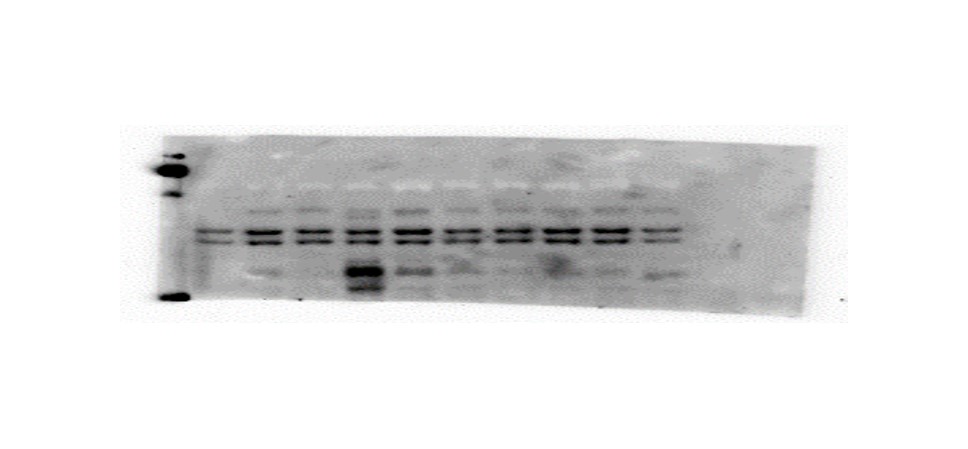

Supplement: Supplementary file 1 [file DataSheet_1.zip › p-ERK.jpg]

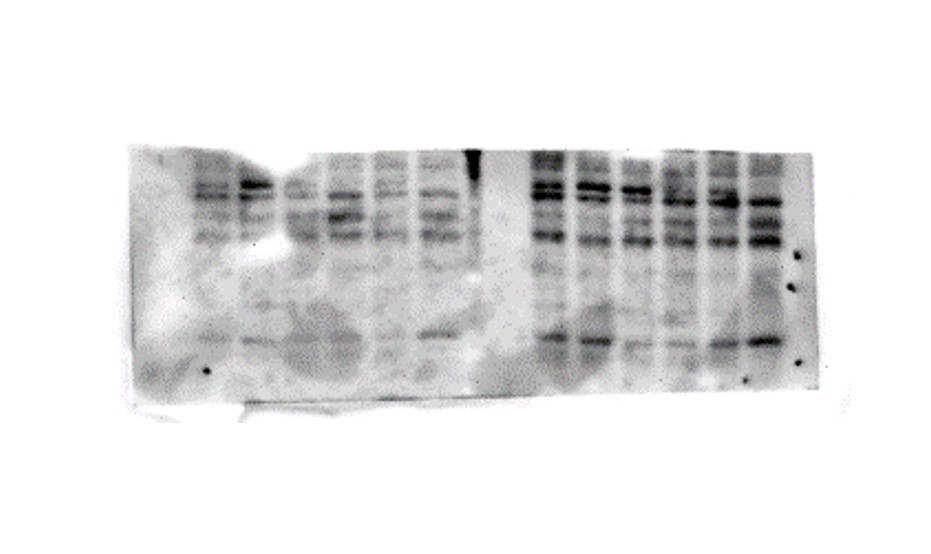

Supplement: Supplementary file 1 [file DataSheet_1.zip › p-MEK.jpg]

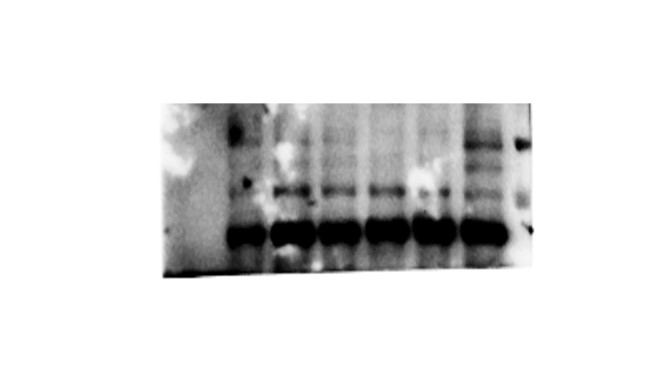

Supplement: Supplementary file 1 [file DataSheet_1.zip › p-Raf-1.jpg]

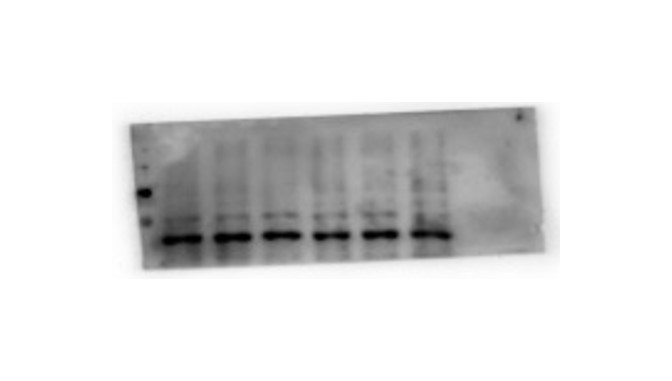

Supplement: Supplementary file 1 [file DataSheet_1.zip › Raf-1.jpg]

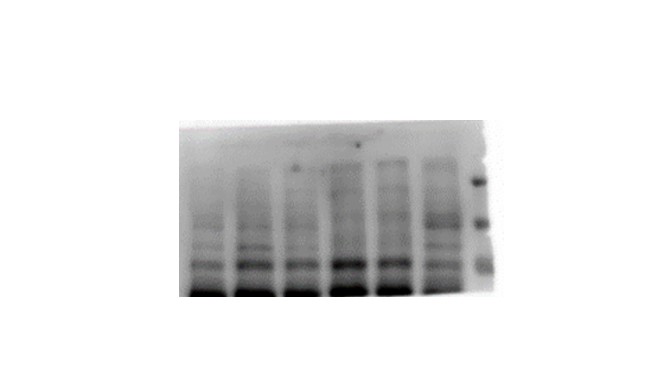

Supplement: Supplementary file 1 [file DataSheet_1.zip › TrkA.jpg]

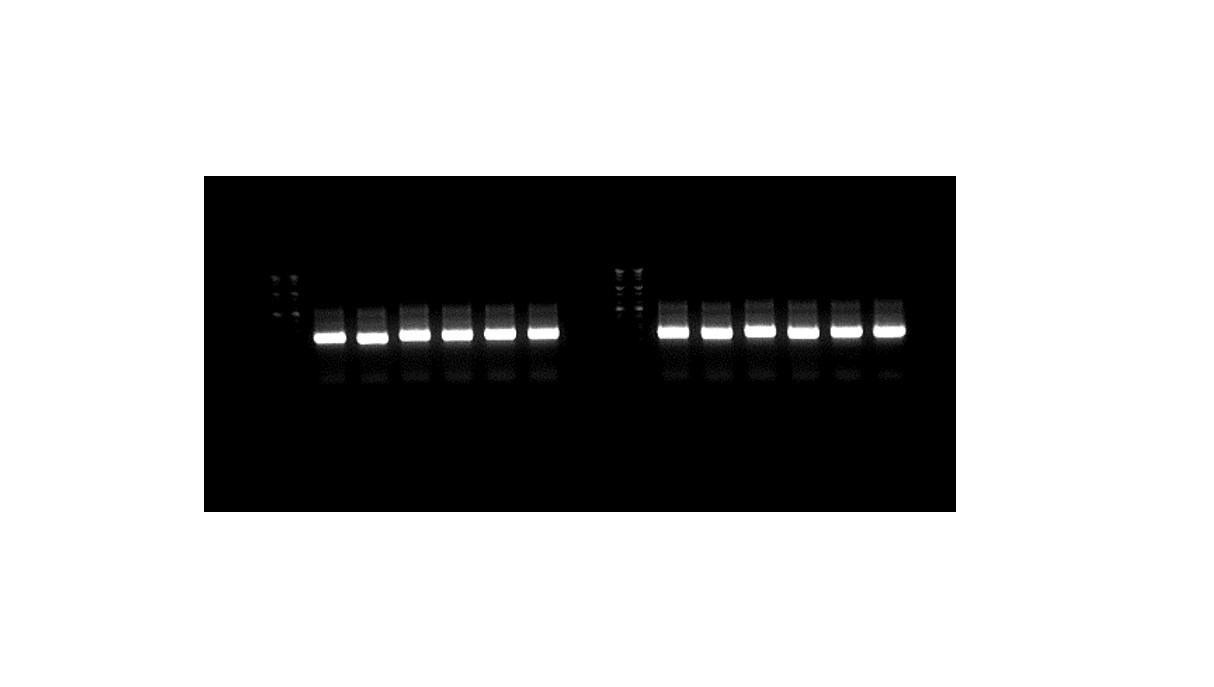

Supplement: Supplementary file 2 [file DataSheet_2.zip › GAPDH.jpg]

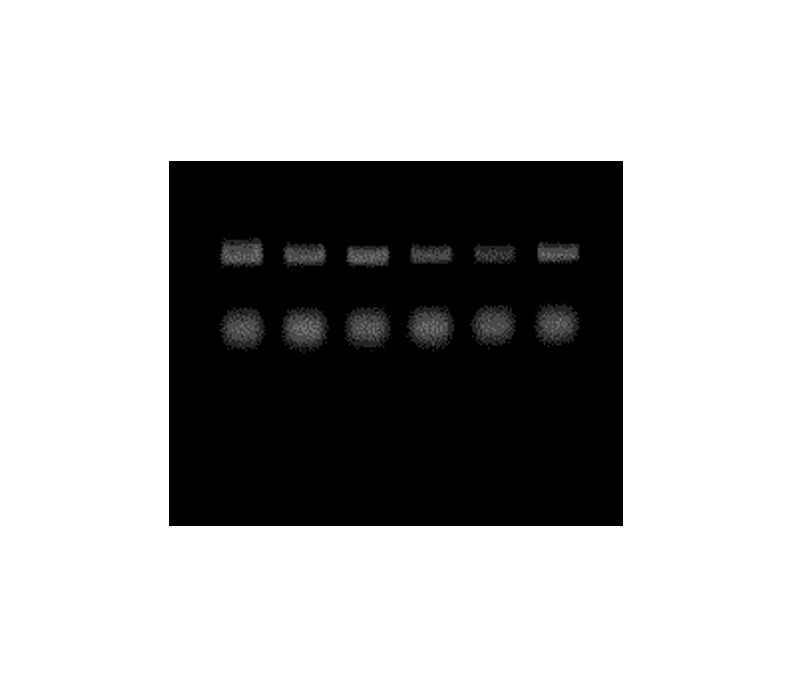

Supplement: Supplementary file 2 [file DataSheet_2.zip › IFN-r.jpg]

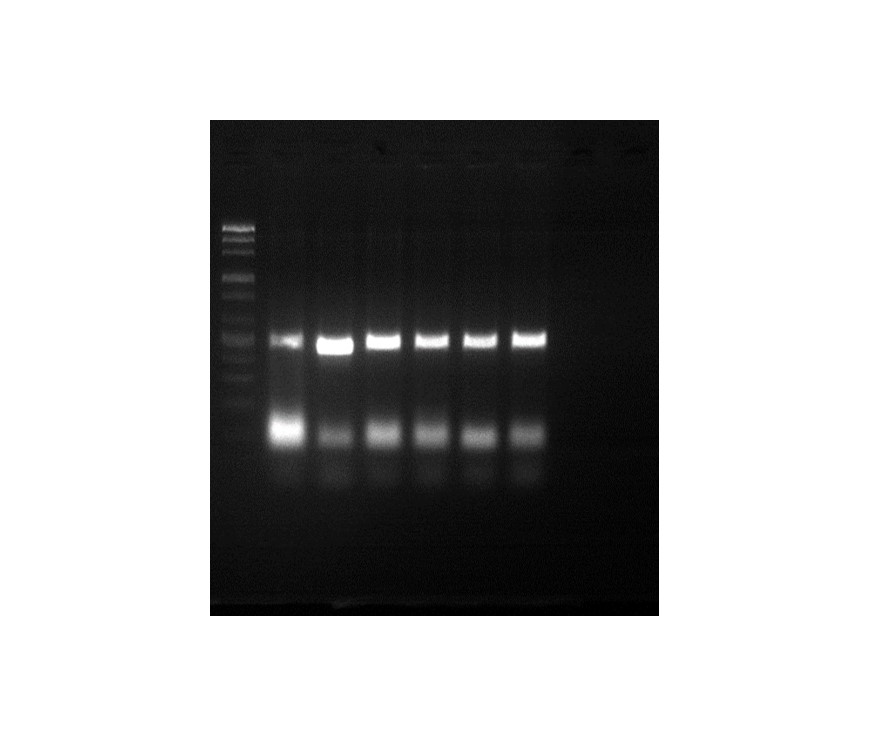

Supplement: Supplementary file 2 [file DataSheet_2.zip › IL-10.jpg]

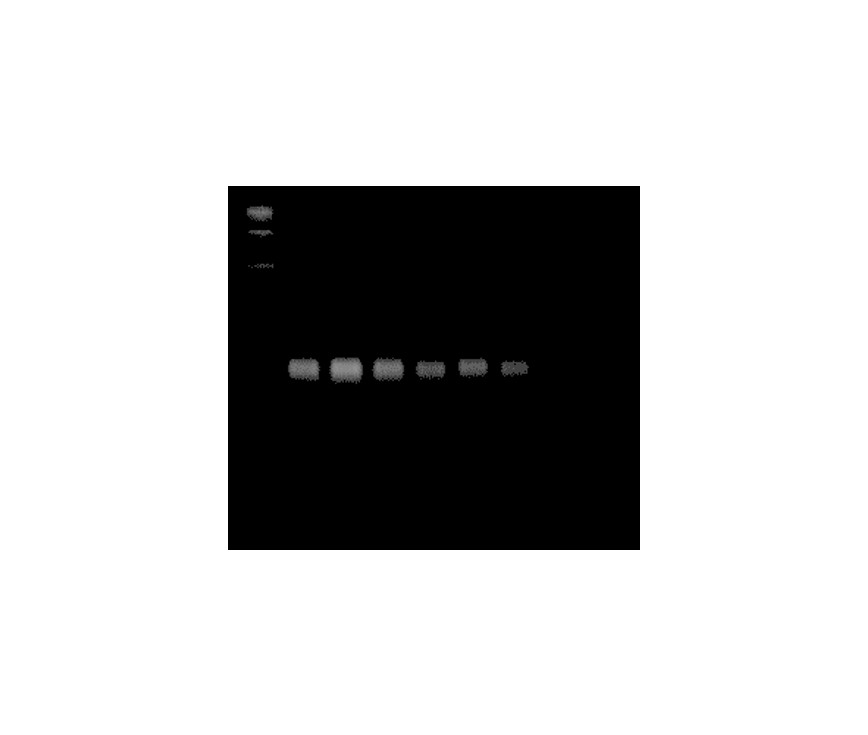

Supplement: Supplementary file 2 [file DataSheet_2.zip › IL-13.jpg]

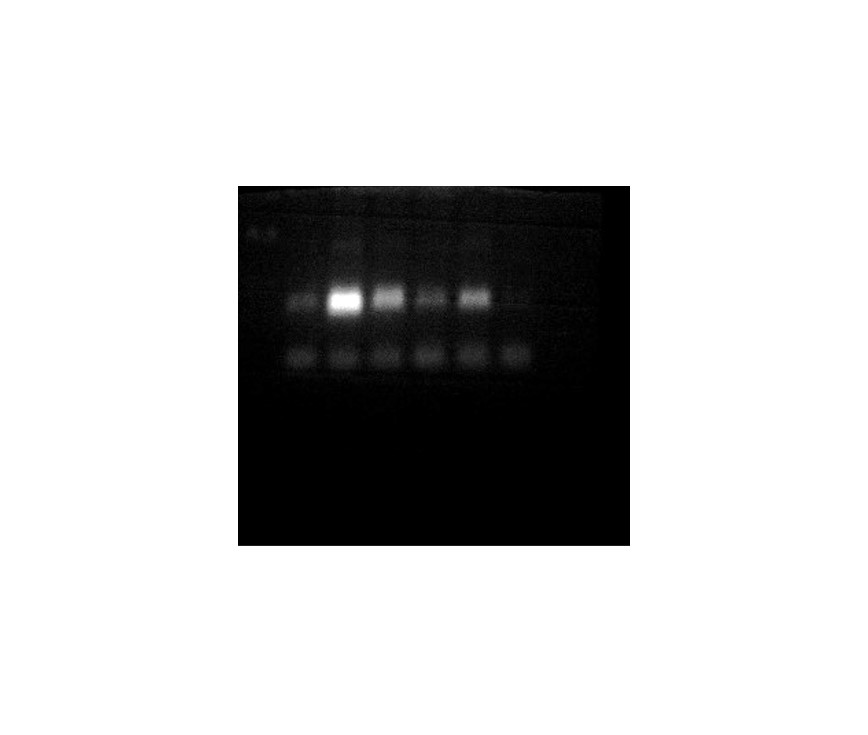

Supplement: Supplementary file 2 [file DataSheet_2.zip › IL-31.jpg]

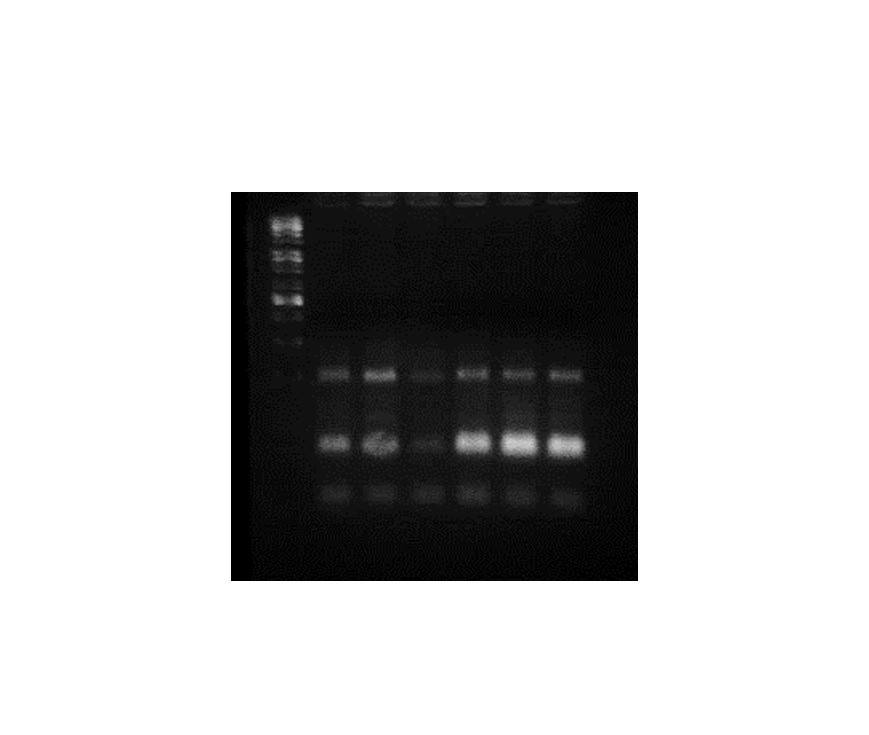

Supplement: Supplementary file 2 [file DataSheet_2.zip › IL-4.jpg]

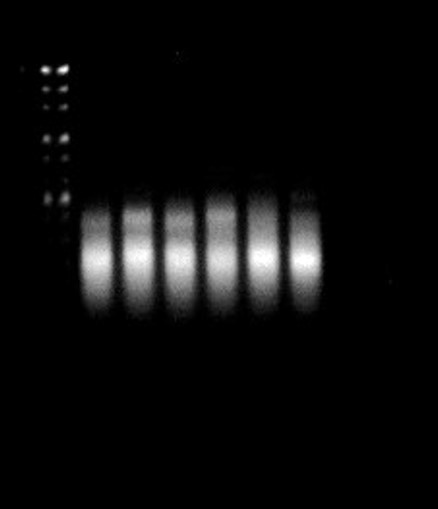

Supplement: Supplementary file 2 [file DataSheet_2.zip › IL-6.jpg]

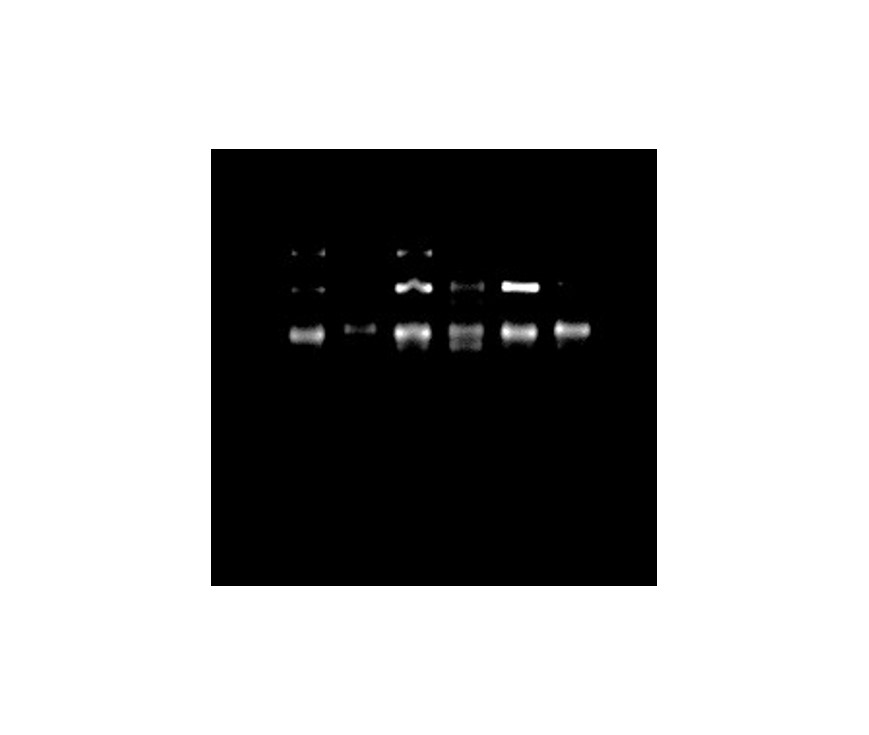

Supplement: Supplementary file 2 [file DataSheet_2.zip › TGF-b.jpg]

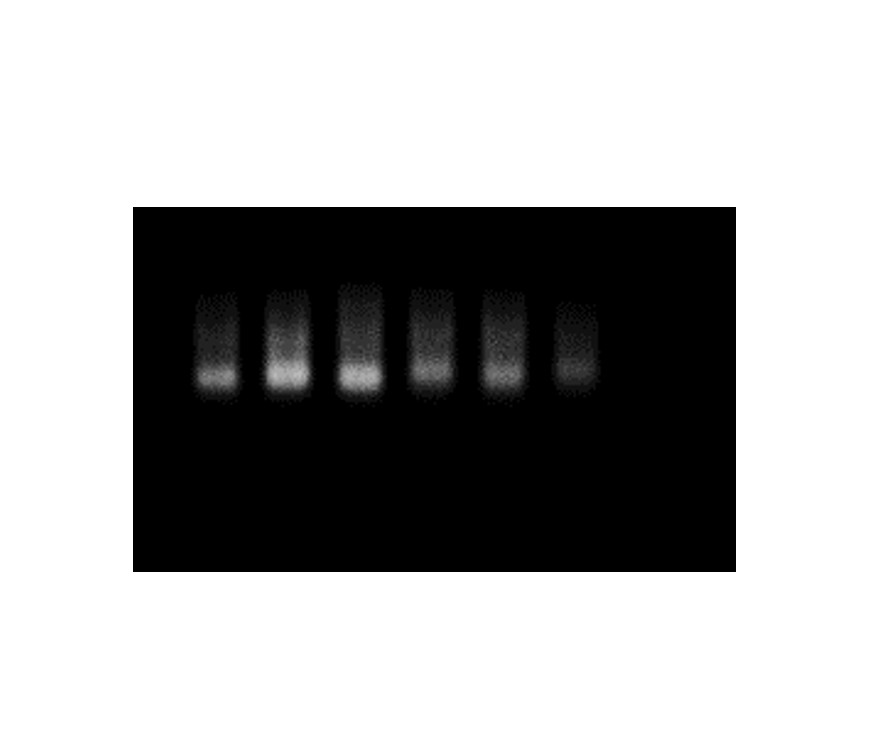

Supplement: Supplementary file 2 [file DataSheet_2.zip › TNF-a.jpg]

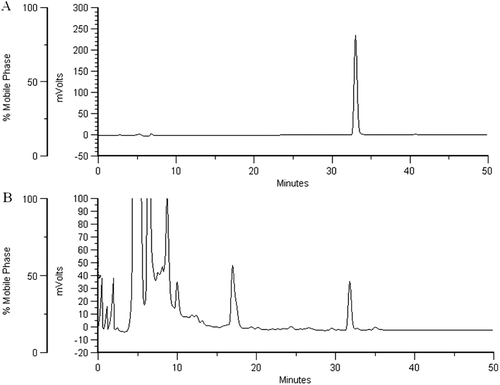

Supplement: Figure S1 — Standardization of Astragalus membranaceus Fisch. ex Bunge using high-performance liquid chromatography (HPLC) systems. HPLC chromatograms of external standard formononetin (A) and AM (B). [file Image_1.tif]

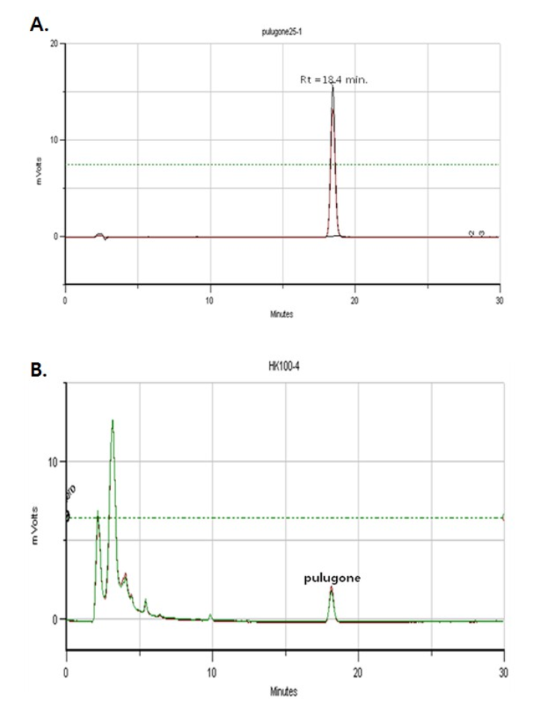

Supplement: Figure S2 — Standardization of Nepeta tenuifolia Benth using high-performance liquid chromatography (HPLC) systems. HPLC chromatograms of external standard pulegone (A) and NT (B). [file Image_2.tif]
